# Prevalence of Temporomandibular Disorder and its association with anxiety in academics: a cross-sectional study

**DOI:** 10.1590/1516-3180.2023.0338.R2.03072024

**Published:** 2024-12-20

**Authors:** Bianca Paludo, Paula Comin Trevizan, Nana Abena Asantewaa Boamah, Lilian Rigo

**Affiliations:** I Dentistry, Atitus Educação, Passo Fundo (RS), Brazil.; II Dentistry, Atitus Educação, Passo Fundo (RS), Brazil.; III Dentistry, Atitus Educação, Passo Fundo (RS), Brazil.; IV Associate Professor, Graduate Program in Dentistry, Atitus Educação, Passo Fundo (RS), Brazil.

**Keywords:** Bruxism, Anxiety, Temporomandibular joint disorders, Students, Anxiety symptoms, Health care field, University, TMD

## Abstract

**BACKGROUND:**

Temporomandibular disorders (TMD) are a major cause of non-dental pain in the oral and facial regions.

**OBJECTIVE:**

This study aimed to determine the prevalence and severity of TMD and anxiety among academics and to investigate the relationship between TMD and its associated factors.

**DESIGN AND SETTING:**

This cross-sectional study included a sample of 295 academics undertaking health courses at a university in Brazil.

**METHODS:**

The Simplified Anamnesis Index and Beck Anxiety Inventory were used to evaluate TMD and assess anxiety, respectively. Data were statistically analyzed using relative and absolute frequencies of variables. In the bivariate analysis, Pearson’s chi-square test was used, and in the multivariate analysis, raw and adjusted binary logistic regressions were used to obtain the odds ratio (OR) and respective 95% confidence intervals. Statistical significance was set at P < 0.05.

**RESULTS:**

The average age of academics was 22.95 (standard deviation ± 6.14) years, predominantly comprising women (82.7%), whites (90.8%), and singles (86.6%). The findings revealed that 81.2% of academics had TMD and 50.5% exhibited symptoms of anxiety. Academics with anxiety were three times more likely to have TMD (OR = 3.6) than those without anxiety.

**CONCLUSION:**

A significant association between anxiety and TMD was observed in academics. The prevalence of TMD was high, with academics with anxiety having a high likely to develop TMD. These findings highlight the importance of addressing mental health concerns in addition to physical health, as they are often related.

## INTRODUCTION

Temporomandibular disorders (TMDs) are a class of musculoskeletal and neuromuscular conditions related to the masticatory muscles, temporomandibular joint (TMJ) complex, and attached bone structures. It results from a combination of factors with varying degrees of psychogenic influence, impairing an individual’s quality of life. Several etiological agents were credited with the cause of this dysfunction, such as occlusal problems, trauma, and emotional stress.^1^ TMDs are highly prevalent, affecting 25% of the population, with a peak incidence from 20 to 40 years of age, and affect women more than men.^2^ The higher prevalence of TMDs among women may be attributed to the fact that women are more likely to seek treatment, indicating a greater tendency for care and attention to health compared to men.^3^

TMDs are the major cause of non-dental pain in the oral and facial regions. The pain can be manifested in the temporomandibular region, face, head, neck, and ears.^4^ The development of pain due to prolonged muscle hyperactivity is mainly related to tissues tension resulting from psychological discomfort, anxiety, or stress. Specifically, the masseter, temporalis, sternocleidomastoid, and trapezius are frequently affected. These muscles react to changes in an individual’s mental state, causing pain associated with psychological disorders and chronic stress.^5^

TMD encompasses conditions affecting the orofacial region, which can be divided into those affecting the masticatory muscles and those affecting the TMJ. The typical features include pain in TMJ, restriction of mandibular movement, and TMJ sounds.^6^

Anxiety is a state of apprehension and anticipation of dangers or unfavorable future events, accompanied by feelings of discomfort, worry, and tension. It is considered pathological when it causes significant suffering or functional impairment.^7^ It involves physiological, mental, and emotional aspects and arises from specific personal traits combined with genetic and social factors, characterized by hypersensitivity to stress and a tendency to experience strong negative emotions such as nervousness, anger, and sadness.^8^

University academics, especially those in their last year of college, are at a higher risk of developing TMDs due to the combined pressures of academic qualification and the uncertainty of future professional activities. The uncertainty of what the next year will be like and where one should settle for their professional development aggravates stress and anxiety levels.^9^ This group was also considered for the study mainly because of high demand for performance during the academic phase, which is a fundamental requirements for becoming future professionals.^10^

## OBJECTIVE

This study aimed to evaluate the prevalence and severity of TMD in academics undertaking health courses (Nursing, Medicine, Dentistry, and Psychology) at a university in southern Brazil. It sought to investigate the association between the presence of TMD and sociodemographic characteristics of the academics (sex, age, and race); days of the week with classes, courses, and the time of the day of the courses; alcohol and coffee consumption; duration of sleep on weekdays and weekends; and use of continuous medication and anxiety.

## METHODS

The Ethics Committee of Research with Human Beings submitted and approved on April 12, 2017 this research under opinion numbers 2,014,448, and CAAE 66373317,6,0000.5319.

### Study design and sample

This study employed a cross-sectional quantitative approach. It included academics who enrolled in health courses (Nursing Medicine, Dentistry, and Psychology) at a university located in a southern Brazilian municipality, were ≥ 17 years of age, met the inclusion criteria, and were eligible to participate in the research. The total number of academics was 1,139, including 113 nursing academics, 335 medical academics, 209 dentists, and 482 psychology academics. Using a 5% margin of error, 90% confidence level, and 50% prevalence of the outcome and adding 15% for potential losses, the calculated sample would comprise 252 academics. However, the final sample size in the present study consisted of 295 academics, which was 43 (17%) academics more than the calculated sample size.

### Procedures for data collection

Data were collected online between March 2021, and September 2021 through a questionnaire available on the Google Forms platform. An invitation with a brief explanation of the research and a link to the online questionnaire was sent to the heads of the nursing, medical, dental, and psychology departments, who then distributed it to the academics via e-mail or WhatsApp, ensuring a broader coverage of participating academics. The study utilized validated scales; anxiety symptoms were evaluated using the Beck Anxiety Inventory questionnaire^11^ and TMD was assessed using the Fonseca Anamnestic Index.^12^ In addition, specific questions were formulated to determine the sociodemographic profile and behavioral characteristics of the academics.

### Variables under study

#### Outcome Variable

#### TMD

The outcome variable of this study was the presence of TMD, evaluated according to the Anamnesis Questionnaire,^12^ which is based on the Helkimo Anamnesis Index.^13^ This is one of the few instruments available in Portuguese that assesses the severity of TMD symptoms. The Simplified Anamnesis Index, developed and validated by Fonseca et al.^12^ consists of 10 questions covering common symptoms, including 1- difficulty opening the mouth; 2- difficulty moving the mandible sideways; 3- muscle pain or tiredness when chewing; 4- frequent headaches; 5- pain at the nape or torticollis; 6- Earaches or joint pain 7- noticeable sounds in the TMJ when chewing or opening the mouth; 8- noticeable habit of teeth clenching or grinding; 9- inability to properly articulate the teeth; 10- considering oneself as a tense or nervous person. Each question has three possible answers with corresponding scores—yes (10), no (0), and sometimes (5). Immediate diagnosis was determined from the total score, which was used for categorizing the severity of TMD as follows: no TMD (0–15 points), mild TMD (20–40 points), moderate TMD (45–65 points), and severe TMD (70–100 points).^14^

The responses were categorized as follows: 1) absence of TMD or without TMD or 2) presence of TMD (mild, moderate, or severe).

### Exposure variables

#### Anxiety

Anxiety was assessed using the Beck Anxiety Inventory, originally developed by Beck et al.^11^ in 1988 and adapted by Cunha.^15^ The inventory consists of 21 items related to the presence of anxiety symptoms, including 1- numbness or tingling; 2- heat sensation; 3- trembling legs; 4- inability to relax; 5- fear that the worst will happen; 6- being stunned or dizzy; 7- heart racing; 8- no balance; 9- feeling terrified; 10- feeling nervous; 11- feeling suffocated; 12- trembling hands; 13- overall trembling; 14- fear of losing control; 15- difficulty breathe; 16- fear of dying; 17- feeling frightened; 18- indigestion or abdominal discomfort; 19- lightheadedness; 20- blushing; and 21- sweating (not due to heat). Items were evaluated on a 4-point scale, with scores ranging from 0 (no symptoms) to 3 (severe symptoms). The total score ranged from 0 to 63. The scores indicate the level of anxiety as follows:^11^,15 0–10 (no anxiety), 11–19 (mild to moderate anxiety), 20–30 (moderate anxiety), and 31–63 (severe anxiety). The inventory was adapted into Portuguese, with data on its accuracy and validity,^15^ maintaining the same scoring system and classifying the intensity levels of anxiety symptoms similar to those of the original version.

Subsequently, for statistical analysis, the answers were categorized as follows: 1) no anxiety (without or with a minimum degree of anxiety) or 2) anxiety (mild, moderate, and severe).

### Sociodemographic and behavioral characteristics

We assessed age (17–20 years or 21–57 years), marital status (married or civil union/single), sex (male or female), race (white or non-white), degree (nursing, medicine, dentistry, psychology), number of class days (1–4 days or 5–7 days), class shifts (morning, afternoon, night or two shifts or three shifts), alcohol consumption (never or a few times a month, 2–4 times a week, or unanswered), coffee consumption (never, one cup a day, or more than three cups a day), and use of continuous medications (yes or no).

### Sleep duration on weekdays and on weekends

Sleep duration on weekdays was determined by asking the academics the following question: “On days you have lectures, what time are you ready to sleep?,” “How many minutes does it take you to fall asleep on school days?,” and “What time do you wake up on school days?.” The duration of sleep on rest days was calculated similarly, only replacing “lecture days” with “rest days.” The number of hours of sleep was calculated by subtracting the time taken to fall asleep (latency time) from the difference between the wake-up time and sleep time. Sleep duration was classified as insufficient if it was < 6 h/day for adults aged 18–64 years.

Subsequently, the answers were categorized as follows: 1) insufficient duration (≤ 6 h) or 2) sufficient duration (> 6 h).

Sleep duration was assessed using a section of the validated Munich Chronotype Questionnaire (MCTQ^PT^),^16^ which has been adapted and validated for the Portuguese language. The MCTQ^PT^ is a complete instrument, which has illustrations that help the respondent to clearly identify the meaning of the questions and make the questionnaire visually attractive.^16^ The questionnaire instructs the interviewee that answers must refer to the past 4 weeks, and although it can be used to define chronotype (biological clock about the light-dark cycle), the questions were only used to characterize the duration and time of sleep latency.

### Data analysis

Data were computed in a Microsoft Office Excel 2016 software database and later exported to the SPSS statistical software (version 20.0; IBM, Armonk, New York) for analysis.

Relative and absolute frequencies of the variables were calculated. Pearson’s chi-square test was used in the bivariate analysis. For multivariate analysis, crude and adjusted binary logistic regressions were used to obtain the odds ratios (OR) and respective 95% confidence intervals (95%CI). All exploratory variables with a P value < 0.20 were included in the crude model, and only those with a P value < 0.05 remained in the adjusted model.

## RESULTS

The average age of the 295 included academics was 22.95 (± 6.14) years, with a sociodemographic profile comprising predominantly women, whites, and singles (82.7%, 90.8%, and 86.6%, respectively). Among the academics, 72.9% attended school 5–7 days per week and 33.9% had three class shifts. Additionally, 15.3% of the academics had insufficient sleep duration on weekdays (< 6 h/day) (**Table 1**).

**Table 1:** Analysis of the variables among academics in a university in Brazil in 2021 (n = 295)

Variables	n	%
**Age (age group)**		
17–20 years	119	40.3
21–57 years old	176	59.7
**Marital status**		
Married/Civil union	39	13.2
Single	256	86.8
**Race**		
White	268	90.8
Non-white	27	9.2
**Number of class days**		
1–4 days	80	27.1
5–7 days	215	72.9
**Class shifts**		
Morning, afternoon, night, or two shifts	195	66.1
Morning, afternoon, or night	100	33.9
**Consumption of alcoholic beverages**		
Never or a few times a month	209	70.8
2–4 times a week	57	19.3
No answer	29	9.8
**Coffee consumption**		
Never or one cup per day	176	59.7
More than three cups per day	119	40.3
**Presence of TMD**		
Neither TMD	53	18
Mild. moderate, or severe	242	82
**Sex**		
Female	244	82.7
Male	51	17.3
**Duration of sleep on weekdays**		
Up to 6h	45	15.3
More than 6 hours	250	84.7
**Sleep duration on rest days**		
Up to 6h	3	1.0
More than 6 hours	292	99.0

TMD = temporomandibular disorder.

Data from academics with TMD are presented in **Table 2**. Among the academics, 43.4% had mild TMD, 27.5% had moderate TMD, and 11.2% had severe TMD. The academics’ data on anxiety are presented in **Table 2**. Notably, 25.8% of the academics had mild to moderate anxiety, 15.3% had moderate anxiety, and 9.5% had severe anxiety. The overall prevalence of anxiety was 50.5% (n = 149).

**Table 2:** Prevalence of temporomandibular disorder and anxiety levels, according to Fonseca’s Anamnesis Index and the Beck Anxiety Inventory, among academics in a university in Brazil in 2021 (n = 295)

	n	%
**TMD**		
Neither TMD	53	18.0
Mild TMD	128	43.4
Moderate TMD	81	27.5
Severe TMD	33	11.2
**Anxiety**		
Absence of symptoms	146	49.5
Mild anxiety	76	25.8
Moderate anxiety	45	15.3
Severe anxiety	28	9.5

TMD = temporomandibular disorder.

Bivariate analysis using Pearson’s chi-square test was performed to examine the association between the presence of TMD and marital status; age; race or ethnicity; alcohol consumption; sex; course; number of class shifts; frequency of coffee consumption per day; use of continuous medication for anxiety, stress, or sleep; sleep duration on working days; and sleep duration on weekends and anxiety. Variables with P values < 0.20 were included in the multivariate model, which included race or ethnicity; sex; age; number of class shifts; continuous medication for anxiety, stress, or sleep; sleep duration on working days; and anxiety. After adjusting for confounding factors in the regression analysis, some variables lost the association with the outcome variable (TMD), which included race or ethnicity; sex; age; number of class shifts; continuous medication for anxiety, stress, or sleep; and sleep duration on working days. Therefore, these variables were excluded from the final model (P > 0.05). In the final adjusted model, the results indicated that academics with anxiety are three times more likely to have TMD (OR = 3.60; 95%CI = 1.79–7.24) compared to those without anxiety (**Table 3**).

**Table 3:** Odds ratio (OR) and confidence intervals (95%CI) between the independent variables and temporomandibular disorder outcome calculated using the binary logistic regression analysis

Variables	Presence of TMD Crude OR (95%CI)	P*	Presence of TMD Adjusted OR (95%CI)	P*
**Sleep duration/working days**				
Enough	1	**0.041** *	1	0.099
Insufficient	2.11(1.03–4.41)		2.01(0.87–4.64)	
**Number of class shifts**				
One or two shifts	1	0.190	1	0.342
Morning, afternoon, or night	1.53(0.79–2.98)		1.41(0.69–2.87)	
**Race**				
White	1	0.100	1	0.359
Non-white	2.08(0.86–5.06)		1.59(0.58–4.32)	
**Sex**				
Female	1	0.056	1	0.158
Male	1.98(0.98–4.02)		1.73(0.80–3.71)	
**Age group**				
17–20 years	1	**0.025** *	1	0.054
21–57 years	2.13(1.10–4.13)		1.99(0.98–4.03)	
**Anxiety**				
No	1	< **0.001** *	1	< **0.001** *
Yes	3.94(2.00–7.7)		3.60(1.79–7.24)	
**Use of sleep medication/stress/anxiety**				
No	1	**0.025** *	1	0.077
Yes	2.26(1.10–4.61)		1.99(0.92–4.27)	

*Wald test – P < 0.05, statistically significant.

OR = odds ratio; CI = confidence interval; TMD = temporomandibular disorder.

Adjusted for race or ethnicity; sex; age; number of class shifts; continuous medication for anxiety, stress, or sleep; duration of sleep; duration of sleep on working days; and anxiety

## DISCUSSION

The present study demonstrated an association between TMD and anxiety, revealing a high prevalence of TMD (81.2%) and anxiety (50.5%) observed among university academics.

The Fonseca Anamnestic Questionnaire was chosen to assess TMD due to its availability in Portuguese, simplicity, and ease of application.^12^ In the literature, various instruments have been investigated for evaluating TMD, including questionnaires, anamnestic and clinical indexes, and diagnostic criteria. The Research Diagnostic Criteria for TMD has been used by several authors.^17, 18, 19, 20, 21, 22, 23^ The Uto-explanatory Screening Questionnaire recommended by the American Academy of Orofacial Pain was used by Manfredi et al.^24^ for clinical examination of TMD. The Craniomandibular Clinical Dysfunction Index, developed by Helkimo^13^ in 1974, is one of the pioneering tools for TMD evaluation. It aims to classify volunteers based on the severity of their TMD clinical signs. The Fonseca Anamnestic Questionnaire, developed in 1994 based on Helkimo’s Anamnestic Index, is one of the few Portuguese instruments used to determine the severity of TMD symptoms. This questionnaire has been tested in patients with TMD and demonstrated a 95% correspondence with the Helkimo Anamnesis Questionnaire.^13^ Researchers evaluating the Fonseca questionnaire, translated into Turkish and Arabic, provide strong evidence of its reliability as a screening tool for TMD.^25,26^ Studies suggested that adapting the questionnaire to include only some of the original questions could potentially increase its reliability. However, the authors emphasized the importance of validation studies to ensure that the new version of the instrument maintains adequate psychometric characteristics.^27^ While the Fonseca Questionnaire is a validated tool widely used for screening TMD, it cannot provide a definitive diagnosis and may not account for all factors that can influence the condition, such as psychosocial or behavioral factors. Therefore, clinical evaluation by a qualified healthcare professional is necessary for a comprehensive and accurate diagnosis of TMD.

The prevalence of TMD in our study was as follows: 18% of the academics did not have TMD, 43.4% had mild TMD, 27.5% had moderate TMD, and 11.2% had severe TMD. The results vary depending on the instruments used. In the present study, 38.7% of the academics required treatment. Among the 295 academics evaluated, 81.2% reported having some degree of TMD. This prevalence was similar to that reported by Garcia et al.,^28^ which was 83.3% among dental academics. Using the same questionnaire, Minghelli et al.^29^ found that 42.4% of university academics from various courses had some degree of TMD. Alamri et al.^30^ reported that 54.2% of academics who studied health courses had TMD. Srivastava et al.^31^ found a 36.9% prevalence among dental academics in Saudi Arabia, similar to the 31.7% prevalence reported in a study including Chinese medical academics. Based on these findings, it can be inferred that academics are likely to feel tense or nervous about various factors such as test evaluations and oral presentations, which may contribute to the development of TMD.

In our study, anxiety was evaluated according to the Beck Anxiety Inventory,^11^ revealing that 50.6% of the academics experienced anxiety, with 15.3% experiencing moderate anxiety and 9.5% experiencing severe anxiety. These findings are consistent with those of Santos et al.,^32^ who reported a 47.4% prevalence of anxiety among academics pursuing health courses, using the same instrument.

A statistically significant association was observed between the presence of TMD and anxiety, with academics experiencing anxiety having three and a half times the likelihood of developing TMD compared to individuals without anxiety. Some studies have reported a significant association between anxiety and TMD with a higher probability of dysfunction occurring in individuals who had anxiety.^33, 34, 35^ However, a different result was observed in a study including dentistry academics, which showed no significant association between TMD and anxiety levels.^9^ In another study, the prevalence of TMD was higher in women than in men.^30^ Bezerra et al.^10^ when conducting a study involving university academics found a higher frequency of TMD in single female individuals, aged 18–22 years. According to Yadav et al.,^36^ the prevalence of TMD was higher in female individuals, accounting for 73% of all cases, and no significant association was found between TMD and sex or age.

Social isolation, changes in routine, and fear of COVID-19 caused an increase in the levels of anxiety, stress, and depression in the general population.^37^ A study conducted by Medeiros et al.^23^ to assess the influence of COVID-19 on TMD symptoms and their consequences reported that during the period of social isolation due to the pandemic, there was an increase in the prevalence of TMD symptoms, depression, and anxiety.

Our study did not find an association between TMD and sleep disorders. However, a previous study evaluated the association between TMD, anxiety (measured using the Beck Anxiety Inventory), and sleep disturbances in dental academics, considering enrollment periods. This study reported a relationship between TMD, sleep disturbance, and enrollment period, revealing that first-year academics had a higher prevalence of moderate to severe anxiety.^38^ In another study, half of the academics exhibited moderate to severe anxiety symptoms, making them more susceptible to poor sleep quality compared to those with lower levels of anxiety.^39^

Studies have evaluated the association between bruxism, TMD, and anxiety. Daytime clenching or grinding has been identified as a significant risk factor for myofascial pain in patients with TMD (OR = 4.9).^40^ Another study explored the association between self-reported sleep bruxism and factors such as age, sex, clinical subtypes of TMD, pain intensity, and degree of chronic pain in patients with TMD. The study found a significant correlation between the presence of sleep bruxism and age < 60 years, female sex, pain intensity, pain interference in their daily activities, and muscle and joint pathology.^41^ Patients with self-reported awake bruxism undergoing orthodontic treatment did not develop TMJ or masticatory muscle pain. However, self-reported awake bruxism was associated with higher levels of anxiety, depression, and a worse quality of life in patients undergoing orthodontic treatment.^38^

The limitations of this study include the online application of the research instrument, which prevented clinical examination to confirm TMD diagnoses. Another limitation was the sampling choice; including academics from other courses beyond the health care area might have yielded different results or variations between undergraduate courses.

The clinical significance of this study lies in identifying parafunctional habits, as the high prevalence indicates that the academic environment can become exhaustive, making academics anxious and influencing the emergence of these habits. Thus, it is necessary to create interventions to address emotional problems affecting academics to prevent the onset of TMD.

However, further studies are required to evaluate their association with other variables.

## CONCLUSION

The results of this study revealed a high prevalence of TMD among academics undertaking health courses at a university in southern Brazil, with the highest prevalence of mild TMD.

Additionally, academics with anxiety were more likely to have TMD compared to those without anxiety.

## References

[1] Al-Groosh DH, Abid M, Saleh AK (2022). The relationship between orthodontic treatment and temporomandibular disorders: A dental specialists’ perspective. Dental Press J Orthod.

[2] Lee YH, Auh QS, An JS, Kim T (2022). Poorer sleep quality in patients with chronic temporomandibular disorders compared to healthy controls. BMC Musculoskelet Disord.

[3] Medeiros SP, Batista AUD, Forte FDS (2011). Prevalence of temporomandibular dysfunction symptoms and oral parafunctional habits in university students. Rev Gaúch Odontol.

[4] Dias WCFGDS, Cavalcanti RVA, Magalhães HV, Pernambuco LA, Alves GÂDS (2022). Effects of photobiomodulation combined with orofacial myofunctional therapy on the quality of life of individuals with temporomandibular disorder. Codas.

[5] Lee YH, Auh QS (2022). Comparison of sleep quality deterioration by subgroup of painful temporomandibular disorder based on diagnostic criteria for temporomandibular disorders. Sci Rep.

[6] Maini K, Dua A (2024). Temporomandibular Syndrome. 2023 Jan 30. In:.

[7] Frota IJ, Fé AACM, Paula FTM, Moura VEGS, Campos EM (2022). Transtornos de ansiedade: histórico, aspectos clínicos e classificações atuais Anxiety disorders: history, clinical features, and current classifications. J Heal Biol Sci.

[8] Abbing A, de Sonneville L, Baars E, Bourne D, Swaab H (2019). Anxiety reduction through art therapy in women. Exploring stress regulation and executive functioning as underlying neurocognitive mechanisms. PLoS One.

[9] Fernandes AÚR, Garcia AR, Zuim PRJ, Cunha LDP, Marchiori AV (2007). Desordem temporomandibular e ansiedade em graduandos de odontologia. Braz Dental Sci.

[10] Bezerra BPN, Arruda AIAM, Farias ABL, et al (2012). Prevalence of temporomandibular joint dysfunction and different levels of anxiety among college students. Rev Dor.

[11] Beck AT, Epstein N, Brown G, Steer RA (1988). An inventory for measuring clinical anxiety: psychometric properties. J Consult Clin Psychol.

[12] Fonseca DM, Bonfante G, Valle AL, Freitas SFT (1994). Diagnóstico pela anamnese da disfunção craniomandibular. RGO.

[13] Helkimo M (1974). Studies on function and dysfunction of the masticatory system. II. Index for anamnestic and clinical dysfunction and occlusal state. Sven Tandlak Tidskr.

[14] Zafar MS, Fareed WM, Taymour N, Khurshid Z, Khan AH (2017). Self-reported frequency of temporomandibular disorders among undergraduate students at Taibah University. J Taibah Univ Med Sci.

[15] Cunha JA (2011). Manual da versão em português das Escalas Beck. Manual.

[16] Reis C, Madeira SG, Lopes LV, Paiva T, Roenneberg T (2020). Validation of the Portuguese Variant of the Munich Chronotype Questionnaire (MCTQ^PT^). Front Physiol.

[17] Fernandes Azevedo AB, Câmara-Souza MB, Dantas IS, Resende CMBM, Barbosa GAS (2018). Relationship between anxiety and temporomandibular disorders in dental students. Cranio.

[18] Wagner BA, Moreira PF (2018). Painful temporomandibular disorder, sleep bruxism, anxiety symptoms and subjective sleep quality among military firefighters with frequent episodic tension-type headache. A controlled study. Arq Neuropsiquiatr.

[19] Simoen L, Van den Berghe L, Jacquet W, Marks L (2020). Depression and anxiety levels in patients with temporomandibular disorders: comparison with the general population. Clin Oral Investig.

[20] Hilgenberg-Sydney PB, Wilhelm JM, Pimentel G, Petterle R, Bonotto D (2020). Prevalence of temporomandibular disorders and anxiety state levels in ballet dancers: a cross-sectional study. J Dance Med Sci.

[21] Bertoli E, de Leeuw R (2016). Prevalence of Suicidal Ideation, Depression, and Anxiety in Chronic Temporomandibular Disorder Patients. J Oral Facial Pain Headache.

[22] Monteiro DR, Zuim PR, Pesqueira AA, Ribeiro Pdo P, Garcia AR (2011). Relationship between anxiety and chronic orofacial pain of temporomandibular disorder in a group of university students. J Prosthodont Res.

[23] Medeiros RA, Vieira DL, Silva EVFD, et al (2020). Prevalence of symptoms of temporomandibular disorders, oral behaviors, anxiety, and depression in Dentistry students during the period of social isolation due to COVID-19. J Appl Oral Sci.

[24] Manfredi AP, Silva AA, Vendite LL (2001). The sensibility appreciation of the questionnaire for selection of orofacial pain and temporomandibular disorders recommended by the American Academy of Orofacial Pain. Rev Bras Otorrinol.

[25] Kaynak BA, Taş S, Salkın Y (2023). The accuracy and reliability of the Turkish version of the Fonseca anamnestic index in temporomandibular disorders. Cranio.

[26] Alyessary AS, Yap AU, Almousawi A (2023). The Arabic Fonseca Anamnestic Index: Psychometric properties and use for screening temporomandibular disorders in prospective orthodontic patients. Cranio.

[27] Campos JADB, Gonçalves DAG, Camparis CM, Speciali JG (2009). Reliability of a questionnaire for diagnosing the severity of temporomandibular disorder. Rev Bras Fisioter.

[28] Garcia RA, Lacerda N, Pereira SLS (1997). Grau de disfunção da ATM e dos Movimentos Mandibulares em adultos jovens. Rev Assoc Paul Cir Dent.

[29] Minghelli B, Morgado M, Caro T (2014). Association of temporomandibular disorder symptoms with anxiety and depression in Portuguese college students. J Oral Sci.

[30] Alamri A, Shahin S, Bakhurji EA, et al (2020). Association of Test Anxiety with Temporomandibular Disorder in Health Professions Students: A Cross-Sectional Study. Int J Dent.

[31] Srivastava KC, Shrivastava D, Khan ZA, et al (2021). Evaluation of temporomandibular disorders among dental students of Saudi Arabia using Diagnostic Criteria for Temporomandibular Disorders (DC/TMD): a cross-sectional study. BMC Oral Health.

[32] Santos dos RM, Simões da MOS (2020). Níveis de ansiedade em alunos concluintes de cursos de saúde. Rev Eletr Farm.

[33] Alahmary AW (2019). Association of Temporomandibular Disorder Symptoms with Anxiety and Depression in Saudi Dental Students. Open Access Maced J Med Sci.

[34] Hekmati A, Mortazavi N, Ozouni-Davaji RB, Vakili M (2022). Personality traits and anxiety in patients with temporomandibular disorders. BMC Psychol.

[35] Motta LJ, Bussadori SK, de Godoy CLH (2015). Temporomandibular Disorder According to the Level of Anxiety in Adolescents. Psic Teor Pesq.

[36] Yadav U, Ahmed J, Ongole R, et al (2020). Influence of psychosocial factors and parafunctional habits in temporomandibular disorders: a cross-sectional study. Perm J.

[37] Rocha JR, Neves MJ, Pinheiro MRR, et al (2021). Alterações psicológicas durante a pandemia por COVID-19 e sua relação com bruxismo e DTM. RSD.

[38] Machado NAG, Costa YM, Quevedo HM, et al (2020). The association of self-reported awake bruxism with anxiety, depression, pain threshold at pressure, pain vigilance, and quality of life in patients undergoing orthodontic treatment. J Appl Oral Sci.

[39] Choueiry N, Salamoun T, Jabbour H, et al (2016). Insomnia and relationship with anxiety in university students: a cross-sectional designed study. PLoS One.

[40] BecMichelotti A, Cioffi I, Festa P, Scala G, Farella M (2010). Oral parafunctions as risk factors for diagnostic TMD subgroups. J Oral Rehabil.

[41] Blanco Aguilera A, Gonzalez Lopez L, Blanco Aguilera E (2014). Relationship between self-reported sleep bruxism and pain in patients with temporomandibular disorders. J Oral Rehabil.

